# Effectiveness of pegylated interferon alfa plus HAART in HIV/HBV treatment-naïve coinfected patients

**DOI:** 10.1186/1758-2652-13-S4-P207

**Published:** 2010-11-08

**Authors:** JA Mata-Marín, CI Arroyo-Anduiza, R Arias-Flores, JE Gaytán-Martínez

**Affiliations:** 1 Hospital de Infectologia, Infectious diseases, Mexico City, Mexico; 2 Hospital de Cardiología/IMSS, Clinical Pathology, Mexico City, Mexico; 3 Hospital de Infectología "La Raza" National Medical Center/IMSS, Clinical Epidemiology, Mexico City, Mexico; 4 Hospital de Infectología "La Raza" National Medical Center/IMSS, Infectious diseases, Mexico City, Mexico

## Background

To our knowledge, the antiviral activity of pegylated interferon alfa plus HAART has not been studied in patients with human immunodeficiency virus type 1 (HIV-1) coinfected with chronic hepatitis B virus (HBV).

## Objective

To evaluate the effectiveness of pegylated interferon alfa plus HAART in HIV/HBV treatment-naïve coinfected patients.

## Methods

We performed a prospective cohort study in HIV/HBV treatment-naïve coinfected patients taking care at "La Raza" National Medical Center, Mexico City. Patients were treated with Efavirenz or Lopinavir-ritonavir, each with tenofovir/emtricitabine plus pegylated interferon alfa-2b (1.5 µg/kg/week) or pegylated interferon alfa-2a (180 µg/week) during 48 weeks. HBV genetic analysis was obtained. The study had a primary measure of effectiveness assessed at 24 and 48 weeks of treatment: suppression of HIV RNA to levels below 50 IU/ml. Secondary endpoints were increased in CD4+ cells count, HBV DNA to levels below 60 IU/ml, HBeAg seroconversion (defined by the loss of HBeAg and the presence of anti-HBe antibody) and HBsAg seroconversion (defined by the loss of HBsAg and the presence of anti-HBs antibody). Cumulative incidence with 95% confidence interval (95%CI) were calculated.

## Results

We enrolled 18 subjects, 1 patient discontinued treatment because adverse events related to PEG-IFN. The mean (± SD) age was 30.3 ± 6.9 years old, all patients were men. The median (interquartile range) basal CD4+ cells count was 112 (61 to 300), RNA HIV 163,000 copies/ml (9,545 to 636,500 copies/ml), DNA HBV 20,200,000 IU/ml (627,500 to 480,500,000 IU/ml). All patients had positive HBeAg and were negative to HDV serology. HBV genotype distribution was H 9 (52%), G 6 (35%), A 1 (6%) and F 1 (6%). Primary endpoint (RNA HIV < 50 copies/ml) was present in 100% of our patient at 24 and 48 weeks; the median increased in CD4+ cells count was 231 cells/ml at 24 weeks and 322 cells/ml at 48 weeks; cumulative incidence of secondary endpoints were: DNA HBV < 60 UI/ml was present in 8 patients [47% CI95% 26-69%)] at 24 weeks and 17 patients (100 %) at 48 weeks; HBeAg seroconversion was in 8 patients [47% CI95% 26-69%)] at 24 weeks and 16 patients [94% CI95% 73-98%)] at 48 weeks, HBsAg seroconversion was in 0 patients (0%) at 24 weeks and 6 patients [35% CI95% 17-58%)] at 48 weeks.

## Conclusions

Pegylated interferon alfa plus HAART were well tolerated and exhibited high viral effectiveness in HIV/HBV treatment-naïve coinfected patients.

